# Enzymatic Stress Responses of *Coreius guichenoti* to Microplastics with Different Particle Sizes

**DOI:** 10.3390/toxics11121022

**Published:** 2023-12-15

**Authors:** Wenqiong Wu, Junqiang Qiu, Yue Lin, Xike Li, Wenjuan Li, Keyi Ma, Yuanliang Duan, Yuanshuai Fu

**Affiliations:** 1 Key Laboratory of Freshwater Aquatic Genetic Resources, Ministry of Agriculture and Rural Affairs, Shanghai Ocean University, Shanghai 201306, China; wuwq0526@126.com (W.W.); jqqiu@shou.edu.cn (J.Q.); wjli@shou.edu.cn (W.L.); 2 Key Laboratory of Exploration and Utilization of Aquatic Genetic Resources, Ministry of Education, Shanghai Ocean University, Shanghai 201306, China; 3 Key Laboratory of East China Sea Fishery Resources Exploitation, Ministry of Agriculture and Rural Affairs, East China Sea Fisheries Research Institute, Chinese Academy of Fishery Sciences, Shanghai 200090, China; kyma1632022@163.com; 4 Fisheries Research Institute, Sichuan Academy of Agricultural Sciences, Chengdu 611731, China

**Keywords:** *Coreius guichenoti*, stress response, stress related enzyme, digestive enzyme, microplastics

## Abstract

The wild population resources of *Coreius guichenoti* have sharply declined in recent decades, and any negative factors may have a significant impact on their survival. In this study, the enzymatic stress responses of *C. guichenoti* to 25 and 48 μm polyethylene fragments were explored for the first time. This was achieved by evaluating the changes in physiological and biochemical indicators of the species in response to the environmental stimuli of microplastics. In this study, we observed an early stress response in the external tissues of *C. guichenoti* following exposure to microplastics. The TP content in skin and muscle and the MDA content in skin, gill and muscle initially showed a significant increase. The skin, gill, and muscle exhibited greater stress responses to M5 particles, whereas M3 particles caused a greater response in the intestine and especially the liver. After the removal of microplastic exposure, the stress state of the *C. guichenoti* would be alleviated in a short period, but it could not fully recover to the pre-exposure level. In summary, microplastics pose a significant threat to *C. guichenoti.* While their negative effects can be alleviated by the removal of microplastics exposure, full recovery does not occur in a short period. Continuous monitoring of microplastics in natural waters and targeted aquatic ecological restoration are essential to ensure the normal growth and reproduction of the wild population of *C. guichenoti*.

## 1. Introduction Test

The *Coreius guichenoti* is an economically important fish species distributed in the main stem and tributaries of the Yangtze River system, including the Minjiang River, Jinsha River, Wujiang River, and others [1,2]. Due to the impact of human activities, such as the construction of a large number of power plant dams [3,4], the wild populations of *C. guichenoti* have sharply declined. In addition to being physical barriers, power generation facilities greatly affect the temperature of the river waters, negatively impacting the growth and reproduction of *C. guichenoti* [5], which thrives in temperatures between 20 and 25 °C [6]. Luo and Wang [7] developed a model that integrated data on water temperature, body weight, and conventional metabolism of *C. guichenoti*. They found that the increase in water temperature caused by the construction of the dam could lead to a significant increase in the energy demand of *C. guichenoti*, posing certain ecological risks. On the 12 December 2007, China officially designated *C. guichenoti* as part of the “List of National Key Protected Aquatic Economic Animal and Plant Resources (First Batch)” [8]. In 2013, *C. guichenoti*, became established as an incidental species in the Yangtze River Basin [9]. Subsequently, on 1 February 2021, China officially listed the wild population of *C. guichenoti* in the “List of Wildlife under Key State Protection”, elevating its status to that of a national secondary protected species [10]. Currently, despite human intervention, the breeding of *C. guichenoti* remains a challenge [11]. Consequently, substantial research investment is crucial for the conservation of the wild population resources of *C. guichenoti*.

Since its invention in 1907, plastic has become an indispensable material in modern society, finding extensive applications in diverse sectors, such as industry and agriculture [12]. Data from the European Plastic Industry Association indicates that global plastic production in 2022 reached approximately 400.3 Mt (https://plasticseurope.org/knowledge-hub/plastics-the-fast-facts-2023/ (accessed on 12 June 2023)). China, a major producer and consumer, accounted for up to 29% of the world’s plastic production [13]. Only 9% of the plastic was recycled after use, with the remainder being discarded as garbage in natural environments [14], thereby contributing significantly to a major global environmental pollution issue. Plastic waste gradually decomposes into small plastic blocks or particles (particle size < 5 mm), commonly known as microplastics, due to the combined effects of physical, chemical, biological and other factors in nature [15,16,17]. Due to its small particle size and strong migratory ability, microplastics can enter various environmental matrices, including the atmosphere, soil, and water [18]. Research has shown that approximately 11% of plastic waste infiltrates aquatic environments [19]. These pollutants not only cause physical harm to aquatic organisms, they also accumulate in the tissues and organs of these organisms, adversely affecting their growth, development, and internal homeostasis [20,21,22]. The microplastics in the Yangtze River primarily consist of polyester, polyethylene, and polypropylene [23,24,25]. The abundance of microplastics increases from upstream to downstream and from the river’s center to its shores, in which lake and reservoir water were higher than that in river water [23,24]. The concentration of microplastics in the surface water of the Yangtze River Basin varies from tens to tens of thousands/m^3^ and continues to rise [23,25]. And the microplastic exposure data from the Yangtze River basin was among the highest reported for rivers and other aquatic systems worldwide, exceeding effect thresholds in some parts of the river [26]. Hence, the potential impact of microplastic pollution on *C. guichenoti* warrants more widespread attention.

Previous studies have demonstrated the infiltration of microplastics into planktonic organisms (cyanobacteria) and invertebrates (mussels and brown shrimp) [27]. The presence of microplastics has also been detected in aquatic vertebrates (fish, turtles, whales, and dolphins), and their transmission through the biological chain [27] poses potential risks to human health. Microplastics exhibit specific toxicity to the digestive, reproductive, and nervous systems of organisms [28]. Microplastics may reduce the lifespan of fish via induction of both suppressed antioxidant system and digestive disturbance as well as hepatic damage [29]. Their impact on the intestinal tract may involve, but is not necessarily limited to, issues such as intestinal obstruction, physical damage, pathological changes in intestinal tissue, behavioral changes, changes in lipid metabolism, and metastasis to the liver [30]. Sequeira et al. [31] found that out of 198 fish species captured across 24 countries, an average of 60% of fish organs contain microplastics. The effects of microplastics on the reproductive system of fish varies among species, mainly resulting in significant reductions in gamete and oocyte quality, fertility, sperm motility, and offspring quality [32]. Exposure to microplastics can induce molecular reactions and histological changes in fish gonads [33]. Huang et al. [34] discovered that microplastics may induce behavioral toxicities in fish through the brain-gut-microbiota axis. Given the complexity of the impacts of microplastics on fish, further research is needed to uncover the underlying mechanisms.

Current research on *C. guichenoti* mainly focused on domestication [35,36], artificial breeding [37,38], and genetic diversity [39,40]. In addition, there have been a small number of studies on gene transcription and cloning [41,42], meat quality [43,44], and other aspects. Research on stress in *C. guichenoti* has primarily focused on parasitic infections and the associated responses triggered by temperature, anesthetics, and other factors. Following parasite infection, various negative changes were observed in the skin, gill, fin, relative fatness, and genetic aspects of *C. guichenoti* [45,46]. Young *C. guichenoti*, in particular, are more susceptible to parasitic infection [45,47]. Scholars have found that the use of anesthetics and low temperatures can alleviate the stress experienced by *C. guichenoti* during transportation [48,49,50,51]. However, there is currently no research on the effects of microplastics on *C. guichenoti*.

In this study, *C. guichenoti* was used as the experimental subject and microplastics of varying particle sizes as the stimulating factor. We examined the dynamic changes in stress substances across different tissues of *C. guichenoti* to analyze the enzymatic stress responses of *C. guichenoti* induced by microplastics. Our findings highlighted the impact of microplastics with varying particle sizes on *C. guichenoti*, shedding light on both the deleterious effects of microplastics on the species and its adaptive response to such exposure. From a physiological perspective, this study contributes valuable insights into the harmful effects of microplastics on *C. guichenoti*, thereby providing data that could contribute to the conservation of the *C. guichenoti* population resources in their natural environment.

## 2. Materials and Methods

### 2.1. Microplastics

Microplastics (polyethylene) were sourced from Huachuang Plastic Raw Material Firm in Zhangmutou Town, Dongguan City, China. These microplastics comprised of two sizes, denoted as M5 and M3, corresponding to 500 mesh (25 μm) and 300 mesh (48 μm), respectively. These materials were in the form of white powders.

### 2.2. Source of Experimental Organism and Experimental Design

In total, 150 artificially bred *C. guichenoti* fry (mean length, 11.79 ± 1.04 cm; mean weight, 12.73 ± 3.72 g) with the same age were obtained from the Fisheries Research Institute, Sichuan Academy of Agricultural Sciences. Then, they were acclimated to laboratory conditions in a circulating aquarium for 7 days prior to the experiment. The water temperature was maintained at 20 ± 1.0 °C with a dissolved oxygen level of more than 8 mg/L. The photoperiod was 12 h:12 h (L/D). All fish were cultured under identical conditions and fed twice daily with aquatic worms at 9:00 and 17:00. Unconsumed feed was removed and 1/3 of the tank water was replaced at 18:00 daily.

After 7 days of acclimatation, fish were exposed to two different conditions: an M5 and M3 particle size with concentrations of 1000 ug/L for the experiment. Each experimental group was set up in triplicate, with 20 fish in each tank. After 7 days of microplastics exposure, the water in the tanks was replaced and the fish were reared for a further 3 days in microplastic free water, with conditions identical to the acclimatation period.

On day 1, 3, 5, 7 and 10 of microplastic exposure, samples were collected, and one fish was collected from each triplicate group at each timepoint. Concurrently, one fish with partial tail fins removed was supplemented in each group to ensure that a total of 20 fish were in each experimental triplicate. In addition, three fish were collected before the start of the microplastic exposure experiment, and the related indicators are detailed in the Supplementary Materials.

### 2.3. Tissue Sample Collection

Before dissection, the fish were first euthanized with a high-dose of tricaine methanesulfonate (MS-222), and then disinfected with 75% alcohol. The skin, gill, muscle, liver, and intestine were collected from each fish. During the collection of skin samples, fish scales and muscle tissues were entirely excised from the skin. During the collection of intestinal samples, the contents of the intestines were gently expressed and the intestines were subsequently washed with phosphate-buffered saline (PBS saline, pH 7.4). 1 g of tissue sample was weighed out, and 0.9% normal saline was added at a ratio of 1: 9 (*w*/*v*). Subsequently, the mixture was homogenized in an ice water bath using a homogenizer. The resulting homogenate was centrifuged at 3500 rpm for 10 min, and the supernatant was collected for enzyme activity analysis.

### 2.4. Enzymatic Activities Analysis

In this study, the enzymatic activities of pepsin, α-amylase and lipase (LPS) in liver and intestine were assessed using the Pepsin Assay Kit, α-Amylase Assay Kit and Lipase Assay Kit (Nanjing Jiancheng Bioengineering Institute, Nanjing, China). Total protein (TP) was determined with the Total Protein Assay kit (Nanjing Jiancheng Bioengineering Institute, China). The enzymatic activities of Acid phosphatase (ACP), alkaline phosphatase (AKP), Peroxidase (POD), Superoxide Dismutase (SOD), Lysozyme (LZM), Glutathione peroxidase (GSH-Px), Glutathione reductase (GR) and Malondialdehyde (MDA) in the skin, gill, muscle, liver, and intestine were assessed using the Acid Phosphatase Assay Kit, Alkaline Phosphatase Assay Kit, Peroxidase Assay Kit, Superoxide Dismutase Assay Kit, Lysozyme Assay Kit, Glutathione peroxidase Assay Kit, Glutathione reductase Assay Kit and Malondialdehyde Assay Kit (Nanjing Jiancheng Bioengineering Institute, Nanjing, China). The assays all were carried out according to the manufacturer’s protocols. In addition, the calculation formulas of these enzyme activities are detailed in the Supplementary Materials.

### 2.5. Data Analysis

Statistical analyses were conducted with Excel and SPSS 19.0 and graphical representations were created using Excel and Adobe Photoshop CC 2018. Prior to formal analysis, normality and equality of variance tests were performed with SPSS software. One-way analysis of variance and multiple comparisons were performed, and the results were expressed as mean ± standard deviation. A significance level of *p* ≤ 0.05 was considered statistically significant.

## 3. Results

### 3.1. Enzymatic Stress Responses of Skin

**Figure 1:**
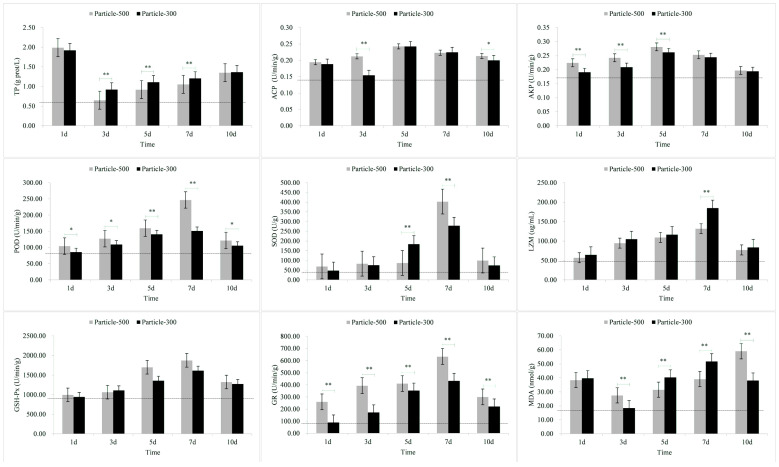
The stress response of enzymes in the skin to microplastic stimuli. Note: Particle-500 represents the microplastics size of 25 μm. Particle-300 represents the microplastics size of 48 μm. The dashed line represents the values of relevant indicators when not stimulated by microplastics. All data are expressed using the mean ± standard deviation, and significant differences from the promoter activity group are indicated by asterisks: * *p* < 0.05, ** *p* < 0.01.

The results of stress response in the skin are shown in Figure 1. The levels of TP and MDA in response to exposure to M5 and M3 microplastics as well as the level of ACP in response to exposure to M3 microplastics rapidly increased within a short period of time and then returned to lower levels. The levels of ACP and AKP exhibited a gradual increase with the duration of exposure, reaching the peak on the 5th day and subsequently declining on the 7th day. The levels of POD, SOD, LZM, GSH-Px and GR also exhibited a gradual increase with the duration of exposure. On the 7th day of exposure, the levels of TP, ACP, LZM and MDA in the M5 group were lower than those in the M3 group, whereas the other indicators exhibited the opposite trend. Following the removal of the microplastics, all the other indicators, except for TP and LZM, were higher in the M5 group compared to the M3 group.

### 3.2. Enzymatic Stress Responses of Gill

**Figure 2:**
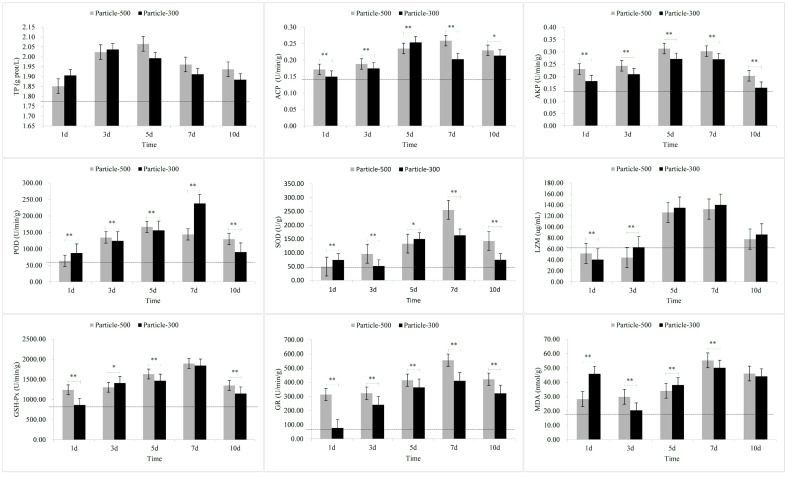
The stress response of enzymes in the gill to microplastic stimuli. Note: Particle-500 represents the microplastics size of 25 μm. Particle-300 represents the microplastics size of 48 μm. The dashed line represents the values of relevant indicators when not stimulated by microplastics. All data are expressed using the mean ± standard deviation, and significant differences from the promoter activity group are indicated by asterisks: * *p* < 0.05, ** *p* < 0.01.

The results of the stress response in the gills are shown in Figure 2. In the M3 group, the levels of SOD and MDA, along with LZM levels in the M5 group, exhibited a rapid increase in a short period followed by a decline. The levels of TP also displayed an increase followed by a decrease over the exposure period. Notably, the peak in the M5 group occurred on the fifth day, while in the M3 group, it occurred on day three. ACP levels in the M5 group exhibited a continuous increase over time, whereas ACP levels in the M3 group showed an initial increase followed by a decline, peaking on the fifth day. The variation pattern of AKP levels in both M5 and M3 groups demonstrated an initial increase followed by a decrease over time, with the peak occurring on the fifth day. POD levels in the M5 group initially increased and then decreased over the exposure period, peaking on the fifth day, while the ACP in the M3 group showed a trend of increasing over time. SOD, LZM, GSH-Px, GR, and MDA gradually increased over time. On the seventh day of the exposure, POD and LZM levels in the M5 group were lower than those in the M3 group, while the other indicators exhibited an opposite pattern. Upon removal of the exposure to microplastics, except for LZM, all other indicators in the M5 group were higher than those in the M3.

### 3.3. Enzymatic Stress Responses of Muscle

**Figure 3:**
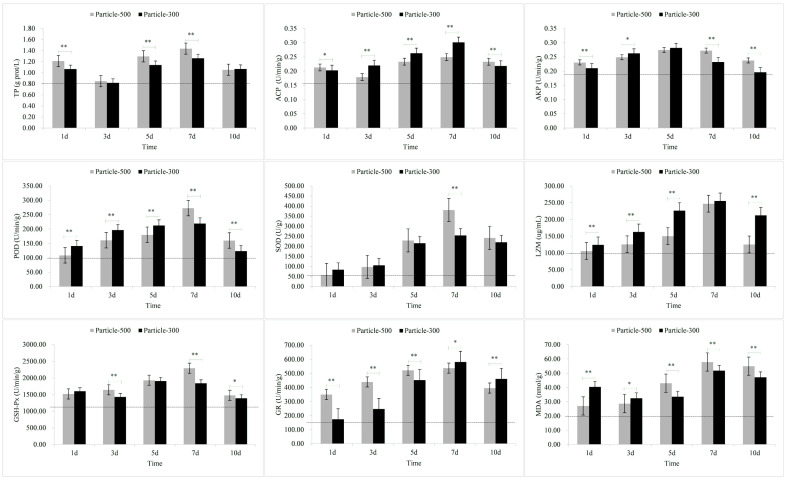
The stress response of enzymes in the muscle to microplastic stimuli. Note: Particle-500 represents the microplastics size of 25 μm. Particle-300 represents the microplastics size of 48 μm. The dashed line represents the values of relevant indicators when not stimulated by microplastics. All data are expressed using the mean ± standard deviation, and significant differences from the promoter activity group are indicated by asterisks: * *p* < 0.05, ** *p* < 0.01.

The results of stress response in muscle are depicted in Figure 3. TP levels in the M5 and M3 group, ACP level in the M5 group, and GSH-Px and MDA levels in the M3 group exhibited a rapid increase followed by a return to lower levels. The variation pattern of AKP showed an initial increase followed by a decrease over time, with the peaks for M5 and M3 observed on the fifth day. GSH-Px levels in the M5 group displayed a continuous increase over time, while in the M3 group, it showed an initial decrease, followed by an increase and eventual decrease, reaching its highest on the fifth day. TP, ACP, POD, SOD, LZM, GR, and MDA levels gradually increased over time. On the seventh day of the experiment, ACP, LZM, and GR levels in the M5 group were lower than those in the M3, while other indicators exhibited the opposite pattern. Upon removing exposure to microplastics, except for TP, LZM, and GR, all other indicators in the M5 group were higher than those in the M3 group.

### 3.4. Enzymatic Stress Responses of Liver

**Figure 4:**
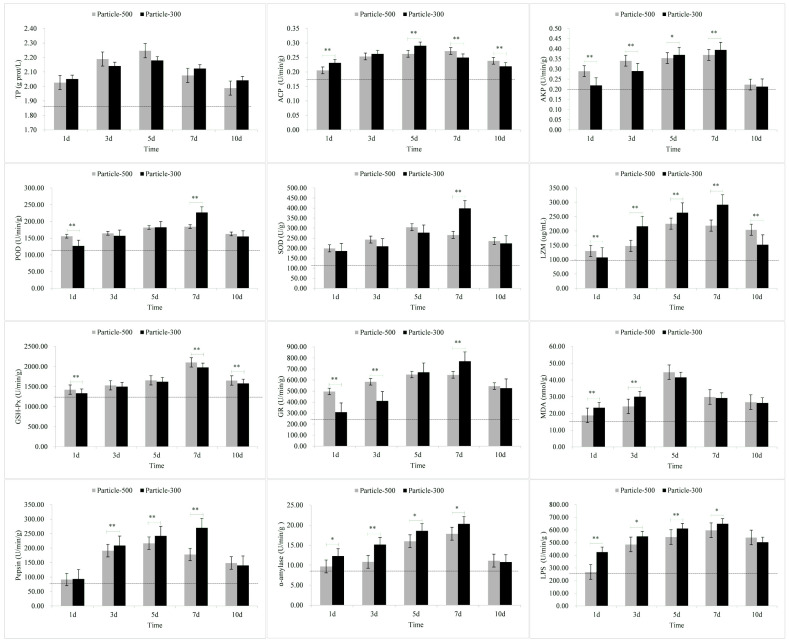
The stress response of enzymes in the liver to microplastic stimuli. Note: Particle-500 represents the microplastics size of 25 μm. Particle-300 represents the microplastics size of 48 μm. The dashed line represents the values of relevant indicators when not stimulated by microplastics. All data are expressed using the mean ± standard deviation, and significant differences from the promoter activity group are indicated by asterisks: * *p* < 0.05, ** *p* < 0.01.

The results of stress response in liver are illustrated in Figure 4. The variation pattern of TP, ACP, and MDA with M5 and M3 exposure exhibited an initial increase followed by a decrease over time, with the highest points observed on the fifth day. SOD, GR, and pepsin levels in the M5 group showed an initial increase followed by a decrease over time, reaching their highest points on the fifth day, while in the M3 group, they exhibited an increasing trend over time. The variation pattern of AKP, POD, LZM, GSH-Px, α-Amylase, and LPS with M5 and M3 exposure showed a trend of increasing over time. On the seventh day of exposure, TP, AKP, POD, SOD, LZM, GR, pepsin, α-Amylase, and LPS levels in the M5 group were lower than those in the M3 group, while other indicators exhibited the opposite pattern. Upon removing exposure to microplastics, except for TP, all other indicators in the M5 group were higher than those in the M3 group.

### 3.5. Stress Adaptive Response of Intestine

**Figure 5:**
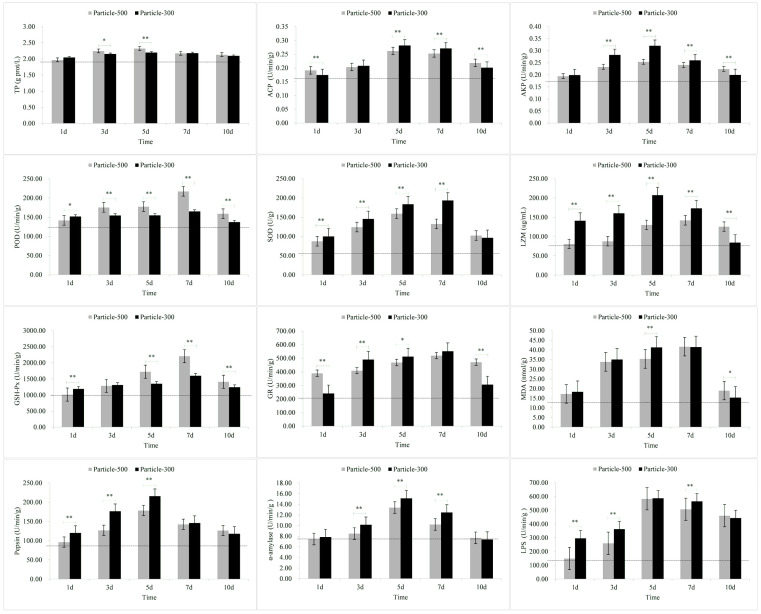
The stress response of enzymes in the intestine to microplastic stimuli. Note: Particle-500 represents the microplastics size of 25 μm. Particle-300 represents the microplastics size of 48 μm. The dashed line represents the values of relevant indicators when not stimulated by microplastics. All data are expressed using the mean ± standard deviation, and significant differences from the promoter activity group are indicated by asterisks: * *p* < 0.05, ** *p* < 0.01.

The results of stress response in intestine are shown in Figure 5. The variation pattern of TP, ACP, AKP, pepsin, α-Amylase, and LPS in the M5 and M3 groups exhibited an initial increase followed by a decrease over time, with the highest points observed on the fifth day. SOD in the M5 group and LZM in the M3 group displayed an initial increase followed by a decrease over time, reaching their highest points on the fifth day, whereas SOD in the M3 group and LZM in the M5 group showed an increasing trend over time. The variation pattern of POD, GSH-Px, GR, and MDA in both M5 and M3 groups showed an increasing trend over time. On the seventh day of exposure, TP, ACP, AKP, SOD, LZM, GR, pepsin, α-Amylase, and LPS levels in the M5 group were lower than those in the M3, while other indicators exhibited the opposite pattern. Upon removal of microplastics exposure, all indicators in the M5 group were higher than those in the M3 group.

### 3.6. Comprehensive Analysis among Different Organizations

After exposure to microplastics, the TP content in skin and muscle and the MDA content in skin, gill and muscle initially showed a significant increase (Figure 1, Figure 2 and Figure 3). The results of the comparative analysis of the effect intensity of microplastics with different particle sizes on *C. guichenoti* are shown in Table 1. In terms of TP, M5 particles had a stronger response from muscle and intestine, whereas M3 particles exhibited a greater influence on the skin. With the ACP stress response indicator, M5 particles caused more reaction from the skin. Additionally, as the experiment progressed, the gill and liver had a stronger response to M5 particles, while the muscle and intestine had a more significant response to M3 particles. From the AKP results, over the course of the exposure, the skin, gill, and muscle had a stronger response to the M5 particles, while the liver and intestines had a greater reaction to the M3 particles. As for the POD indicator, M5 particles exhibited a stronger initial effect on the skin, and exerted a progressively pronounced effect on the muscle and intestine over the course of the study, while M3 particles elicited a stronger reaction from the gill and liver over time. In terms of the SOD levels, the muscle had a greater reaction to the M5 particles, while M3 particles had a stronger effect on the liver and intestine. Over the course of the experiment, the SOD levels in the skin and gills had a bigger response to M5 particles. In terms of the LZM response indicator, there was a greater immediate response from the skin, muscle, and intestine to the M3 particles. Additionally, M3 had a stronger response the gill and liver as the study progressed. For the GSH-Px stress response indicator, the muscle and liver exhibited a stronger initial reaction to the M3 particles, whereas over the course of the study, the gill and intestine had a stronger response. In terms of GR, M5 particles elicited a stronger reaction from the skin and gill, while M3 particles had a greater response from the muscle, liver and intestine. In terms of MDA, M3 particles exhibited a significant impact on the liver and intestine. Additionally, over the course of exposure, the gill and muscle had a stronger response to M5 particles whereas the skin had a stronger response to M3 particles. For the pepsin, *α*-amylase and LPS indicators, M3 particles had a greater response from the liver and intestine. The stress response to M5 particles in *C. guichenoti* was more cumulative and pronounced in the skin, gills, and muscles. In contrast, the response to M3 particles by *C. guichenoti* was more direct and pronounced in the intestine, but also exhibited cumulative effects in the liver. Specifically, M5 particles elicited greater stress responses in the skin, gill, and muscle, while M3 particles caused stronger stress responses, especially in the liver and intestine. After removing microplastics from the water, the residual M3 particles in the fish body caused an increased response from LZM and GR in the muscle, while all other indicators have opposite patterns in different organs, totaling 25 indicator-organs.

## 4. Discussion

As a nationally designated secondary protected species, the wild population of *C. guichenoti* is constantly influenced by human activities, necessitating additional efforts to ensure the completion of its normal growth and reproduction cycle [6]. Furthermore, the presence of toxic and harmful substances such as microplastics and heavy metals [52], poses long-term and cumulative risks to the survival of *C. guichenoti*. Microplastics, in particular, have strong potential for accumulation in both the environment and living organisms [53]. Consequently, studying the adverse effects of microplastics on *C. guichenoti* holds great scientific importance.

To our knowledge, this study is the first to investigate the connections between the stress responses and microplastics in *C. guichenoti*. The selected enzymes in this study are commonly recognized as indicators of stress [54,55,56,57,58], effectively demonstrating the stress response of *C. guichenoti* after being exposed to microplastics.

Skin is the organ that is the most directly exposed to various environmental stimuli, such as microplastics and hypoxia [59,60]. As such, its response to stressors may be more heightened compared to other tissues. Upon stimulation, the skin produces a significant quantity of mucus, with protein being the primary component [61,62]. In this study, the levels of TP and MDA in both the M5 and M3 groups, as well as ACP levels in the M3 group were significantly higher on the first day compared to the third day, in particular for TP and MDA. This may be attributed to *C. guichenoti* initially producing abundant mucus and superoxide radicals in response to exposure to microplastics, resulting in the increased production of MDA. This phenomenon was proven by Kim et al. [63], who found that microplastics also induced an imbalance in reactive oxygen species production and antioxidant capacity, causing oxidative damage. In addition to the skin, gills, being in direct and prolonged contact with the aquatic environment, may promptly exhibit stress reactions when exposed to environmental stimuli. In this study, exposure to M3 particles resulted in significantly higher levels of SOD and MDA on the first day compared to the third day. Furthermore, in this study, the changes in stress-related indicators of muscle were similar to those observed in the skin, possibly owing to the proximity of muscle to the skin. In the M3 group, the levels of GSH-Px in the muscle was significantly higher on the first day compared to the third day. Compared to skin, gill, and muscle tissues, the liver and intestine exhibited a more cumulative response to microplastics, indicating an increase in stress responses with prolonged exposure time. However, with prolonged exposure to negative stimuli, the homeostasis in the fish body may become imbalanced [64], and the stress-related indicators may cease to increase [65]. In our study, the values of indicators such as ACP in the liver and AKP in the intestine began to decrease on the fifth day after exposure to microplastics. These research results suggest that tissues on the body surface of *C. guichenoti* may be more susceptible to damage after exposure to microplastics.

The intake of microplastics by fish could be active or passive [66,67]. The amount of time these microplastics are retained in the fish body may be related to their particle size [68]. Current research on the interaction between fish and microplastics are mainly focused on the gastrointestinal tract [66,68]. In this study, tissues exposed to the external environment, such as the skin and gill, as well as internal tissues including the muscle, liver, and intestine, were selected to investigate the stress effects of microplastics with different particle sizes on *C. guichenoti*. The removal rate of microplastics from the fish intestines, particularly in fish with stomachs, increased with larger particle sizes [68]. Naidoo et al. [69] studied the accumulation of microplastics in the intestines of fish along the South African coastline and found that the particle sizes of microplastics ranged from 0.1 to 4.8 mm. However, there were few large particles, and the average particle size of microplastics was about 0.89 ± 0.77 mm. The statistical results in Table 1 suggests that the external organs of the *C. guichenoti* were more susceptible to small particle microplastics, whereas the internal organs exhibited greater susceptibility to large particles. This could be due to smaller particles easily infiltrating the surface tissues through passive contact, while the larger particles had to be actively ingested, leading to a greater influx into the internal organs such as the intestine. The process of entering the intestinal tissue resulted in stronger stimulation to the intestine.

In this study, the stress response of *C. guichenoti* after removal of microplastic exposure, was lower in the M3 group compared to the M5 group, which could be attributed to the faster excretion rate of larger microplastics from the body. Kaloyianni et al. [70] exposed *Danio rerio* and *Perca fluviatilis* to microplastics and found that the gills seemed to be more responsive to microplastics compared to the liver, which was similar to the results of our study. In this study, the MDA levels in the gill ranged from 40 to 140 mmol/g, while in the liver it was only 20 to 40 mmol/g. In addition, in this study, after being stimulated by microplastics, the TP content in the skin and MDA content in the skin and gill significantly increased on the first day, and decreased on the third day. This response could be due to the two organs being the first to be exposed to microplastics. Taken together, the results indicate that different tissues in the body *C. guichenoti* respond differently to exposure to microplastics due to mode of exposure and particle size.

In addition to stress-related parameters, we also studied three major digestive enzymes of *C. guichenoti*. Wen et al. [71] observed that microplastics exerted a greater effect compared to elevated temperatures on the predatory performance, digestion and energy production of *Symphysodon aequifasciatus*. This impact was associated with an increase in the enzyme activities of the related digestive enzymes. Trestrail et al. [72] found that microplastics could increase cellulase and protease activities in the digestive gland of *Mytilus galloprovincialis*. In this study, pepsin, *α*-amylase and LPS also showed similar changes. There was a significant increase in enzyme activities when stimulated by microplastics, potentially leading to increased energy consumption in *C. guichenoti*. The presence of microplastics significantly stimulated the specific activity of *α*-amylase and non-specific esterases in *Coregonus peled* Gmelin larvae after 24 h, and this was similar to the results of this study [73]. After the removal from exposure to microplastics, the digestive system of fish will gradually recover to some extent [74], which is consistent with our results. In our study, the digestive enzyme activities of *C. guichenoti* decreased, to some extent, on the third day after removal of microplastic exposure; however, they did not revert to the pre-exposure levels.

## 5. Conclusions

As toxic substances capable of persistent accumulation in fish bodies and transmission through the biological chain, microplastics pose a significant threat to the already scarce wild population of *C. guichenoti*. This study was the first to explore the stress responses of different tissues of *C. guichenoti* to exposed microplastics of different particle sizes. It also quantifies the impact of microplastics on *C. guichenoti* using multiple parameter indicators. This study revealed that the surface tissues of *C. guichenoti* may exhibit earlier and increased susceptibility to the negative effects of microplastics, with these effects being reversible. Hence, linking the environmental management of microplastics with the protection of the *C. guichenoti* population is essential for ensuring the sustainability of their resources.

## Figures and Tables

**Table 1:** Comparative analysis of the effect intensity of microplastics with different particle sizes on *C. guichenoti*.

Tissue	Stress Response Indicators	M5	M3
Skin	TP	−	+
	ACP	+	−
	AKP	+’	−
	POD	+	−
	SOD	+’	−
	LZM	−	+
	GSH-Px	−	−
	GR	+	−
	MDA	−	+’
Gill	TP	−	−
	ACP	+’	−
	AKP	+’	−
	POD	−	+’
	SOD	+’	−
	LZM	−	+’
	GSH-Px	+’	−
	GR	+	−
	MDA	+’	−
Muscle	TP	+	−
	ACP	−	+’
	AKP	+’	−
	POD	+’	−
	SOD	+	−
	LZM	−	+
	GSH-Px	−	+
	GR	−	+
	MDA	+’	−
Liver	TP	−	−
	ACP	+’	
	AKP	−	+’
	POD	−	+’
	SOD	−	+
	LZM	−	+’
	GSH-Px	−	+
	GR	−	+
	MDA	−	+
	pepsin	−	+
	*α*-amylase	−	+
	LPS	−	+
Iintestine	TP	+	−
	ACP	−	+’
	AKP	−	+’
	POD	+’	−
	SOD	−	+
	LZM	−	+
	GSH-Px	+’	−
	GR	−	+
	MDA	−	+
	pepsin	−	+
	*α*-amylase	−	+
	LPS	−	+

Note: The data is sourced from the Figure 1, Figure 2, Figure 3, Figure 4 and Figure 5. These include comparative data that all reached significance. The “+” indicates that M5 or M3 has a greater effect on the *C. guichenoti*. The “+’” indicates that over time, M5/M3 has a greater effect on the *C. guichenoti*. The ‘−’ indicates that the data has not reached significance or there is no relevant data.

## Data Availability

The datasets generated and/or analyzed during the current study are not publicly available owing to security protocols and privacy regulations, but they may be made available on reasonable request to the corresponding author.

## Supplementary Materials

The following supporting information can be downloaded at: https://www.mdpi.com/article/10.3390/toxics11121022/s1, Table S1: The stress related indicators and digestive enzymes of *Coreius guichenoti* before microplastics stimulation; Formula for calculating enzyme activity.
