# Supplementary material for: Enzymatic Stress Responses of Coreius guichenoti to Microplastics with Different Particle Sizes
Source: Toxics. 2023 Dec 15;11(12):1022. doi: 10.3390/toxics11121022 (PMC10747963; doi:10.3390/toxics11121022)
Supplement: Supplementary file 1 [file toxics-11-01022-s001.zip › toxics-2723278-supplementary.pdf]

Table S1 The stress related indicators and digestive enzymes of *Coreius guichenoti* before microplastics stimulation

Tissue	total protein (g L^{-1})	ACP (U min^{-1} g^{-1} prot)	AKP (U min^{-1} g^{-1} prot)	POD (U min^{-1} g^{-1} prot)	SOD (U g^{-1} prot)	GPx (U min^{-1} g^{-1} prot)	GR (U min^{-1} g^{-1} prot)	MDA (mmol g^{-1} prot)	LZM (μg ml^{-1})	Protease (U min^{-1} g^{-1} prot)	α-amylase (U min^{-1} g^{-1} prot)	Lipase (U min^{-1} g^{-1} prot)
Skin	0.63 ± 0.05	0.14 ± 0.00	0.17 ± 0.00	82.37 ± 1.01	40.81 ± 1.42	918.75 ± 44.27	78.55 ± 3.39	17.02 ± 0.86	47.94 ± 1.14	na	na	na
Gill	1.77 ± 0.05	0.14 ± 0.00	0.14 ± 0.00	59.90 ± 2.55	45.61 ± 1.06	819.63 ± 26.66	69.75 ± 2.66	17.93 ± 0.48	36.91 ± 1.45	na	na	na
Muscle	0.81 ± 0.00	0.16 ± 0.01	0.19 ± 0.00	99.07 ± 2.55	55.51 ± 3.72	1126.73 ± 49.12	151.44 ± 5.83	19.68 ± 2.62	99.71 ± 2.56	na	na	na
Liver	1.86 ± 0.02	0.18 ± 0.01	0.20 ± 0.00	116.72 ± 10.97	108.30 ± 4.00	1243.09 ± 26.72	245.98 ± 12.75	15.28 ± 1.34	97.64 ± 2.97	80.04 ± 1.51	8.50 ± 0.43	262.37 ± 6.69
Intestine	1.93 ± 0.08	0.16 ± 0.00	0.17 ± 0.00	124.75 ± 5.79	57.59 ± 3.96	990.31 ± 19.46	209.34 ± 7.03	12.87 ± 2.07	76.61 ± 0.43	86.20 ± 2.87	6.80 ± 0.31	133.32 ± 11.16

Note: “na” represents there was no relevant data. $n = 3$.

Formula for calculating enzyme activity

Total protein contents (TP, g L^{-1}) = (OD*a* - OD*b*) / (OD*c* - OD*b*) * A * B

Note: OD*a* represents the absorbance value of the sample. OD*b* represents the absorbance value of the blank group. OD*c* represents the absorbance value of the standard product. A represents the standard product concentration (0.563 g/L). B represents the sample dilution ratio.

ACP (U min^{-1} g^{-1} prot) = 0.1 * (OD*a* – OD*b* + 0.0018) / W * D

Note: OD*a* represents the absorbance value of the sample. OD*b* represents the absorbance value of the blank control. W represents the sample weight. D represents the sample dilution ratio.

AKP ($U\ min^{-1}\ g^{-1}$ prot) = 0.128 * (ODa – ODb - 0.0019) / W * D

Note: ODa represents the absorbance value of the sample. ODb represents the absorbance value of the blank control. W represents the sample weight. D represents the sample dilution ratio.

POD ($U\ min^{-1}\ g^{-1}$ prot) = 100 * (ODa – ODb) / W * D

Note: ODa represents the absorbance value of the sample at "n + 1" mins. ODb represents the absorbance value of the sample at "n" mins. W represents the sample weight. D represents the sample dilution ratio.

SOD ($U\ g^{-1}$ prot) = 10 * A / (1- A) / W *D; A (%) = [(ODc - ODd) - (ODa - ODb)]/ (ODc - ODd) * 100%

Note: A represents the inhibition rate of SOD. W represents the sample weight. D represents the sample dilution ratio. ODa represents the absorbance value of the sample. ODb represents the absorbance value of the blank in the experimental group. ODc represents the absorbance value of the control group. ODd represents the absorbance value of the blank in control group.

GPx ($U\ min^{-1}\ g^{-1}$ prot) = 683.7 * (ODa – ODb + 0.0103) / W * D

Note: ODa represents the absorbance value of the sample. ODb represents the absorbance value of the blank control. W represents the sample weight. D represents the sample dilution ratio.

GR ($U\ min^{-1}\ g^{-1}$ prot) = 73.5 * (ODa – ODb) / W * D

Note: ODa represents the absorbance value of the sample at "n + 10" mins. ODb represents the absorbance value of the sample at "n" mins. W represents the sample weight. D represents the sample dilution ratio.

MDA ($mmol\ g^{-1}$ prot) = 32.3 * (ODa – ODb) / W * D

Note: ODa represents the absorbance value of the sample in 532 nm. ODb represents the absorbance value of the sample in 600 nm. W represents the sample weight. D represents the sample dilution ratio.

LZM (μg ml^{-1}) = 10 * (OD*a* – OD*b*) / (OD*c* – OD*d*) * D

Note: OD*a* represents the absorbance value of the sample at "n + 2.5" mins. OD*b* represents the absorbance value of the sample at "n" mins. OD*c* represents the absorbance value of the standard sample at "n + 2.5" mins. OD*d* represents the absorbance value of the standard sample at "n" mins. D represents the sample dilution ratio.

Protease (U min^{-1} g^{-1} prot) = 35 * (OD*a* – OD*b*) / (OD*c* – OD*d*) / W * D

Note: OD*a* represents the absorbance value of the sample. OD*b* represents the absorbance value of the blank in the experimental group. OD*c* represents the absorbance value of the control group. OD*d* represents the absorbance value of the blank in control group. W represents the sample weight. D represents the sample dilution ratio.

α-amylase (U min^{-1} g^{-1} prot) = 6.88 * (OD*a* – OD*b* + 0.0039) / W * D

Note: OD*a* represents the absorbance value of the sample. OD*b* represents the absorbance value of the blank control. W represents the sample weight. D represents the sample dilution ratio.

Lipase (U min^{-1} g^{-1} prot) = 307.7 * (OD*a* – OD*b* + 0.0003) / W * D

Note: OD*a* represents the absorbance value of the sample at "n + 10" mins. OD*b* represents the absorbance value of the sample at "n" mins. W represents the sample weight. D represents the sample dilution ratio.
